# Observations on the Specific Properties of Diseases and Medicines; with Cases, Illustrative of the Power of Bark in the Cure of Periodical Diseases

**Published:** 1826-08

**Authors:** John Allan

**Affiliations:** Member of the Royal Colleges of Surgeons of London and Edinburgh.


*Observations on the Specific Properties of Diseases and Medicines; with Cases, illustrative of the Power of Bark in the Cure of Periodical Diseases.* By John Allan, Member of the Royal Colleges of Surgeons of London and Edinburgh.

It is, unfortunately, in but a few examples, among the numerous catalogue of diseases incident to mankind, that remedies capable of directly controlling or extinguishing morbid processes have as yet been discovered; and it is probable that the powers of the few, of which the existence has been observed, are not so often nor so effectually taken advantage of as they might be, if the minds of those who are engaged in the practice of medicine were sufficiently impressed with the importance of carefully discriminating between those minute shades of difference in the features of diseases, and the operation of remedies, which, though they may seem unimportant to a careless observer, often constitute essential distinctions. There is, perhaps, no department of medicine in which superior judgment is required, than in the detection of such distinctions,—in the selection of the fit remedies,—and in determining the proper periods and doses for their successful exhibition. For, to practise medicine with eminent success, it is not sufficient to be able merely to determine whether depletion or tonics be required; though even this is often a question of much difficulty. The accomplished physician ought to be capable of weighing with accuracy all the circumstances of the case, and of selecting, out of whatever class of medicines may be indicated, the individual article precisely adapted to the peculiarities of his patient's case.

In the great majority of diseases, the assistance of the practitioner extends only to reducing or restraining excessive action of the vascular system, soothing nervous irritation, and aiding or regulating secreting organs in the discharge of their functions. Blood-letting, when properly instituted as to time and quantity, may frequently arrest the progress of a disease, such as pneumonia or hepatitis; but it possesses not the property of a specific agent, nor can it be said to operate otherwise than by reducing the action of the heart and arteries below that degree which is essential to the support of the disease in question.

In many diseases, however, as phthisis, scrofula, syphilis, small-pox, measles, &c. it is impossible to induce resolution, or to arrest the course of the disease, by any means simply reductive in their operation: on the contrary, in some of these diseases,—as phthisis, for example,—when the strength of the general system is reduced to the lowest ebb compatible with the continuance of the vital functions, the morbid process going on in the affected organ is more active than ever. This proves that it is only by means of specific remedies that art can ever accomplish the radical cure of such diseases.

It must be admitted that, in a certain sense, every medicine is a specific: that is to say, every remedy possesses a property of producing a peculiar effect on the body, which cannot be precisely produced by any other article, even of the same class. Thus, opium and wine are capable of producing effects of an analogous nature; but no quantity that can be given of the one is capable of producing precisely the same effects, or of operating in exactly the same manner, as a certain dose of the other. Many medicines, in like manner, possess in common the power of exciting the peristaltic motion of the intestines, and of increasing the quantity of secretion poured into the alimentary canal; but it is universally known that, among the class of purgatives, there is as great a diversity in their modes of operating as in their names.

The specific properties of diseases are, perhaps, no less numerous and distinct than those of remedies. Even in those complaints which are most analogous to each other, or of which the mutual resemblance is greatest, each possesses distinctive properties, or combinations of symptoms, which are peculiar to it alone, and by due attention to which it may be recognised and discriminated from all others. The importance of an exact knowledge of the specific powers of medicines, for directly counteracting or neutralising the morbid influences of diseases,—as of mercury in syphilis, and of sulphur in scabies,—is universally admitted; and the vast number of medicines, in favour of which unfounded pretensions to the possession of specific properties have been put forth by interested individuals, ought not to prevent us from endeavouring to ascertain the precise limits and extent of the powers inherent in those, the virtues of which are generally admitted.

The principal object of the present paper is to lay before the profession some facts, which seem to illustrate, in a very striking manner, the property which bark has long been known to possess as an antidote to periodical diseases. The peculiar mode in which it will be seen that the bark was administered, may also be worthy of notice and of further trial.

### I. *Case of severe periodical Pain in the Ankle, cured by Bark.*

A shopkeeper, about thirty years of age, applied to me, about the beginning of February 1821, on account of a painful affection of one ankle. There was no swelling nor discoloration; but every night, after going to bed, it became affected with a severe pain, which entirely prevented sleep for many hours. He could not remain in bed long, but used frequently to get up and walk about in his shirt, being somewhat easier while doing so than when warm in bed. Towards three o'clock every morning, the pain attained an agonising degree of severity. After that period it remitted, and towards daylight he used to obtain a little sleep. Occasionally a considerable time would elapse after waking, before he could bear to place his foot firmly on the floor; nor could he bear friction upon the ankle when the pain was at the height, although, when it first began to affect him, he fancied that some slight relief was derived from frictions with warm flour, or with the hand alone. During the day he pursued his business without feeling any pain whatever. Nine months before this period, the pain had commenced in both ancles, but had soon become entirely fixed in the left; and, as it seemed uniformly to increase in severity, and threatened the ruin of his health, he felt the greatest apprehensions as to the result.

I commenced the cure by purging him with calomel and the pilula colocynthidis composita, for three successive days; after which he took *ten grains* of powdered cinchona every three hours, the bowels being kept open by smaller doses of the purgatives. After he had taken twelve of the powders, the pain ceased: I advised him, however, to take twelve more. The pain never returned, but, about ten days after, he was suddenly seized, in the night, with an ungovernable fit of frenzy. He was a man of sober habits, and, on the strictest inquiry, I could not discover that he had deviated from them on this occasion. I advised him to take some purgative medicines, and to observe a spare diet.

During four years after this, I had occasionally opportunities of seeing him, and he never had the slightest return either of the pain or mental affection. went off with a bang.

II. *Case of severe periodical Pain of the Right Leg, cured by Bark.*

A footman, twenty-eight years of age, applied to me, on the 19th of April, 1823, on account of severe pains affecting both legs, and more particularly along the rotular aspect of the tibiæ. In November 1822, five months before I saw him, he had been fifteen days, during very boisterous and wet weather, on his passage from Aberdeen to London, during which time he had been deprived of regular rest, and exposed to wet and fatigue. From that period he began to suffer severe pain along the fore part of the left leg, which continued for two months, and then gradually extended to the whole of that limb. At this time the right leg likewise began to be affected in the same manner; and, when he applied to me, he still had much pain in the left, but it was most severe in the right. He described the pain as observing regular diurnal periods, coming on every afternoon at five o'clock, and increasing in severity during the night, so as entirely to prevent sleep. He declared positively that, during the five months that had elapsed since he landed from the vessel, he had never known the comfort of an hour's sleep; and that he was generally compelled to get out of bed ten or a dozen times every night, to walk about his room, so intolerable was the pain as soon as he became warm. He was considerably emaciated and very pale, with an expression of anguish imprinted on his countenance. His tongue was very much furred, although, according to his own statement, his appetite continued good, and his bowels regular.

I commenced, as in the former case, with purging, by means of compound colocynth pill and calomel, with the addition of a grain of antimonial powder to each pill. After several days purging, the pains continued unabated. I then directed *ten grains* of pulvis cinchonæ to be taken every two hours. He called upon me twice afterwards, at intervals of about eight days. The first time he was not entirely free from the pains, but they were much less severe, and allowed him to enjoy the refreshment of sleep. He had acquired a fresh complexion, and had manifestly gained flesh. The last time he called was to inform me that he was entirely well, which was sufficiently attested by his improved appearance.

III. *Case of severe periodical Pain in the Legs, cured by Bark.*

A married woman, thirty-seven years of age, and mother of seven children, applied to me, in February 1826, on account of severe pains in her legs. In May 1823, she had made a voyage of five days from Penzance to London, during which she had been exposed to cold and wet, and had been deprived of rest. The first night after her arrival in London, she had been seized with shiverings and with pains in her shoulders; and, for twelve months after that period, she had been constantly affected with such pains, but not always in the same part. At one period, and for some months, her hearing had been very imperfect, and her vision double. About February 1824, her left leg had become affected with severe pains, extending from the knee to the instep; and three months afterwards, the right leg had become affected in the same manner. Ever since that period her complaints had wholly centered in the legs; but such had been their severity, that she had never, during the two years, been able to pass a whole night in bed, or to obtain any sleep before daylight, as the pains usually became agonising as soon as she got warm in bed. For the pains in her legs she had attended two Dispensaries successively, for a considerable time, with little or no relief. Leeches had been repeatedly applied, and a variety of medicines taken, under the direction of one gentleman, during a period of thirteen weeks. For a considerable period before applying to me, she had desisted from seeking further assistance, and had been in the habit of attempting to deaden her sense of pain by taking laudanum daily. She was thin and pale, and her countenance was strongly expressive of habitual suffering. Her skin was dry, but not hot; her tongue somewhat furred.

After purging for three days with colocynth and calomel, I directed her to take *five grains* of powder of bark, with the same quantity of carbonate of soda, every two hours, in a little tepid water. The second night after beginning the use of these powders, (having also left off her laudanum from the time she first consulted me,) she rested without any pain; but also (probably from long habit) without any sleep. She declared that she had not known so comfortable a night for two years. Having omitted taking the powders for a week, she felt a slight return of the pains; but the renewed use of the powders speedily relieved her again, and I advised her to take three or four daily for some weeks afterwards. Since that time I have not heard of her.

The great similarity of these cases, and the efficacy of the bark, has led me to think that a report of the facts might draw to the subject the attention of those who have had more extensive opportunities than myself of treating such cases. I am aware that nothing is more likely to ruin the reputation of any medicine than praise unduly lavished upon it. Many are apt, in consequence of such encomiums, to try it in cases apparently similar, but in fact essentially different from those to which it is actually applicable, and, not finding the beneficial effect which they had been led to expect from its use, they are ready to infer, not that they had given the medicine in a case unsuited to its specific properties, but that it really did not deserve the encomiums which had been bestowed upon it. I think it, therefore, of some importance to state, that in cases of pain in the extremities, however intractable the complaint may have proved to other remedies, I have not seen benefit obtained from bark, *unless the pain had a periodical character.* The following case affords an illustration of this fact.

IV. *Case of severe Neuralgic Pain, which was not relieved by Bark, but cured by Subcarbonate of Iron.*

About the same time that the third case was under my care, a gentleman, about thirty-eight years of age, of a nervous temperament, and rather fond of his glass, who had for many years been subject to occasional fits of syncope, and to a constant nervous tremor, affecting chiefly his hands, was seized with pain of a peculiar character in the region of the right scapula. It was not increased by pressure, nor was there any swelling or other visible change. It was generally troublesome during the night, though not invariably so. It was occasionally entirely suspended for several hours at a time, without any known cause. After the use of some purgatives combined with colchicum, the disease seemed rather to increase than diminish.

Reflecting on the influence of the bark in the cases related above, I was induced to try it in the present instance; but it was not of the smallest avail, and, after three or four days' use, was left off.

The pain, some days afterwards, became more severe than ever, and the fleshy part of the forearm of the same side became affected in a similar manner. The parts at length became somewhat tender when pressed. A medical gentleman, who saw the patient on one occasion when suffering under a severe paroxysm of the pain near the spine of the scapula, was of opinion that a deep-seated abscess was forming in that situation. Leeches and fomentations to the part afforded some relief, but it was only of short duration. The patient observed, about this period, that the pain was apparently induced by directing his attention to it, (as by talking about it;) and that it was invariably brought on by shaving. At length, the mere appearance of the hair-dresser, who attended every morning to shave him, became a certain signal for its return. Some nights, however, were passed with little or no pain, but never twenty-four hours without some paroxysms of it.

I gave the precipitated subcarbonate of iron, beginning with a scruple every six hours, increased after a few days to the extent of a drachm every four hours. Under this remedy the disease gradually declined, and soon completely disappeared; while the patient was surprised to find that the nervous tremors of his hands were very much lessened, and at times entirely suspended. His general health and strength were remarkably improved.

There seems very little to discriminate between this case and the preceding ones, except the invariable nocturnal aggravation of the pain in the latter, and the irregular and apparently capricious returns of it in the former. Yet I apprehend that the subcarbonate of iron, by which the disease in the last case was so satisfactorily subdued, might have been given without avail for the relief of either of the preceding. I am not inclined to place much confidence in any explanation that has yet been suggested of the manner in which the specific effects of medicines are produced, and am by no means disposed to indulge in speculations upon the subject. It seems, however, not unreasonable to believe that bark exerts its influence through the medium of the nervous system. The paroxysm of an intermittent fever has sometimes been prevented by a large dose, taken within half an hour of the accession of the expected fit; a space of time that would seem too short to admit of the digestion and absorption of the medicine.

With respect to the cases related above, there is, perhaps, no particular in their history more remarkable than the smallness of the doses in which the bark was given; whereas, according to the doctrines generally admitted respecting this medicine, it should be given as frequently, and in as large doses, as the stomach will bear. In the first of the cases just related, a painful disease of nine months' duration was completely subdued by twelve doses, containing altogether only two drachms of bark; a quantity much less than has often been taken in a single dose for ague. If it be correct to assume that bark, when taken into the stomach, has a direct influence, through the medium of that organ, upon the nervous system,—it may perhaps account for the power of small doses of the medicine to assume further, that the repetition of its impulses a considerable number of times within a given space of time,—that is, within the period intervening between the attacks of two paroxysms of the periodical pain, in cases of the kind related above,—contributes more effectually to secure the curative effects of the remedy, than the impulse produced by a smaller number of large doses taken within the same time.

Whether this explanation be admitted or not, is a matter of little moment. The merit of being the first to ascertain that bark may be given, in certain cases, with as much, or greater, efficacy in minute than in large doses,—or rather, that it may be exhibited with the most beneficial effects in small doses, where large ones would be wholly inadmissible, —belongs, as far as I know, to Mr. Wardrop. In a paper on Rheumatic Ophthalmia, published in the tenth volume of the Medico-Chirurgical Transactions, he states that "bark seems to possess as specific an effect in this disease as in ague;" and that the doses he generally gave were from five to eight grains every two hours. It was from having observed the success of this practice in some cases of rheumatic ophthalmia under Mr. Wardrop's care, that I was induced to give the bark in small doses. I have since had occasion to exhibit it in several cases of that disease, and have had the satisfaction of seeing it produce an immediate change, from a prolonged state of pain and misery scarcely to be endured, to one of composure and comfort. The following case may serve as an example:

V. *Case of Rheumatic Ophthalmia, cured by small doses of Bark.*

A poor woman, of a thin, spare habit, about fifty years of age, had suffered for several weeks from a most painful affection of one eye. The pain darted through one side of the head. During the day it remitted in a considerable degree, but every evening it recurred in such severe paroxysms as totally to deprive her of sleep. A variety of means were ineffectually tried; general and local bleeding, active purging, repeated blisters on the nape of the neck, behind the ears, on the temples, and on the forehead, afforded little or no relief. Opium and conium, in large doses, aggravated the pain in the head, without suspending the progress of the disease affecting the eye. When I first examined this, at the request of the surgeon under whose care she was, it presented extreme vascularity of the tunica conjunctiva, frequent gushing out of hot tears, an incipient superficial ulceration of the cornea, and a decided inactivity of the iris, indicating that that part participated in the inflammation. As soon as the iris had appeared to be implicated in the disease, the patient had been placed under the full influence of calomel and opium, but without the least benefit; or, rather, with the effect of aggravating her sufferings.

From the appearance of the eye, I suspected the disease to be of a rheumatic nature; and this suspicion was confirmed by the history of the case, and more especially by the fact that the patient had formerly been subject to rheumatism in her limbs, but had been wholly exempt from that form of the disease since the affection of her eye commenced.

I recommended that she should take ten grains of bark, with five grains of subcarbonate of soda, in a little water, every three hours. At the same time she was directed to take one grain of calomel, with four grains of pilula colocynthidis composita, every night. The patient began taking the bark about two o'clock in the afternoon; and that very night she enjoyed more rest and comfort than she had known since the commencement of the attack. The recovery of her health kept pace with the abatement of the local inflammation, and was both speedy and perfect. A thin speck, however, remained upon that part of the cornea where the ulceration had commenced.

In this case the attack of the disease had been sudden, and apparently without any cause specifically determining it to the eye; but experience enables me to say that any cause of local irritation affecting that organ is very apt to act as a predisposing cause of rheumatic ophthalmia, when a patient is exposed to any of the ordinary exciting causes of rheumatism.

It is now admitted, I believe, even by the warmest eulogists of the specific virtues of calomel and opium in iritis, that there are certain cases in which that disease will not yield to these remedies. The case related above, in which the inflammation being of the rheumatic kind, and extending from the sclerotic coat, its proper seat, to the adjacent parts, and not having originated in the iris, would seem to be one of a class of cases in which calomel and opium ought to be avoided as injurious.

